# Direct new oral anticoagulants: follow-up, guidelines and bleeding complications in general practice—a survey of Swiss general internal medicine practitioners

**DOI:** 10.1186/s40064-016-3722-z

**Published:** 2016-11-29

**Authors:** Thomas C. Sauter, Carlo Melis, Wolf E. Hautz, Meret E. Ricklin, Aristomenis K. Exadaktylos

**Affiliations:** Department of Emergency Medicine, Inselspital, Bern University Hospital, University of Bern, Freiburgstrasse, 3010 Bern, Switzerland

**Keywords:** Apixaban, Bleeding event, Dabigatran, Everyday general practice, Real-life, Rivaroxaban

## Abstract

**Background:**

The present study investigated how much Swiss general internal medicine practitioners (GPs) know about new direct oral anticoagulants (NOACs), particularly the relevant guidelines, follow-up tests, dosing adjustments, indications and complications. We conducted a paper-based survey of GPs, performed in Bern, Switzerland. Our questionnaire assessed the physicians’ preference for NOACs rather than vitamin K antagonists (VKA), prevalence and choice of NOAC, clinical follow-up including follow-up blood testing, and bleeding complications.

**Results:**

53 GPs participated in our pilot investigation. They treated 32.7% ± 19 of their patients requiring oral anticoagulation with NOACs. New patients who had started oral anticoagulation received NOACs from 49 GPs (92.5%) but most GPs would not switch patients from existing VKA therapy to NOACs. Clinical controls are scheduled by a majority of GPs (67.9%) at least every 3 months; creatinine and haemoglobin are monitored by most GPs (51 (96.2%) and 39 (73.6%), respectively). In the preceding 2 years, GPs had seen 1.9 ± 2.87 bleeding complications in patients with NOACs. 0.5 ± 0.95 (range 0–5) of these required hospital treatment.

**Conclusion:**

NOACs are broadly accepted by investigated Swiss GPs as the first choice for patients newly requiring oral anticoagulation. This was in preference to VKAs and especially if recommended by a haematologist or cardiologist. As, in our population, only about two-thirds of GPs adhere to recommendations on clinical and blood test follow-ups, further efforts to implement follow-up guidelines seem necessary. Further research in a large representative GP population is recommended; this should compare NOACs and VKAs. Bleeding complications were rare in our population and could mostly be handled without hospital admission.

## Background

New direct oral anticoagulants (NOACs) have been introduced in recent years and this type of oral anticoagulation is of increasing importance in everyday clinical work, in both emergency departments (EDs) and general practice. In addition to the studies for initial approval, plenty of data have been published on the safety and efficacy of NOACs (Connolly et al. 2009; Granger et al. 2011; Patel et al. 2011). On the other hand, few studies report on the use of these drugs in hospitals or general practices.

Lee et al. (2012) assessed the representativeness of the registration studies RE-LY, ARISTOTLE and ROCKET-AF and concluded that they were not equally representative of the real-life UK population with atrial fibrillation. Such problems with trial generalizability are recognised and have to be taken into account when trying to transfer trial results into general practice (Weiss et al. 2008).

In a preliminary study, Southworth et al. (2013) compared patients on dabigatran with those on warfarin and found similar incidences of gastrointestinal and intracerebral bleeding events. Recent observational data from our university emergency department have confirmed the low incidence of serious bleeding events. We found that epistaxis, haematomas and gastrointestinal bleeding are the most frequent bleeding complications under the new drugs (Sauter et al. 2016).

Further real life observational data—external validation rather than registration studies—are essential, as adherence to guidelines in everyday practice is unclear with respect to follow-up tests, dosing adjustments and indications. It might e.g. be speculated that patients with NOACS gain less attention in follow-up than do patients on vitamin K antagonists (VKAs), as INR testing is not required for NOACs. A recent investigation of the prescription and use of NOACs in the USA showed that 39% of all patients received an inappropriate dose of NOACs. Moreover, 6% of patients reported that they missed about one dose each week (Simon et al. 2015).

We therefore aimed to investigate the real-life handling and complications of patients treated with NOACs, as encountered in everyday general practice and as influenced by general internal medicine practitioners’ (GPs’) awareness of NOAC therapy and follow-up guidelines.

## Methods

We conducted a paper-based survey of GPs attending a GP emergency medicine congress at Inselspital, Bern University Hospital, Switzerland. Demographic data on age, gender, speciality and professional experience were collected.

Our questionnaire consisted of four topics (Questionnaire is available on request.):

- We asked GPs whether they preferred NOACs to VKAs and, if so, why. We also inquired how many of their patients were on NOACs in comparison to VKAs and whether the GPs switched from VKAs to NOACs for anticoagulation.
- We examined the choice of a NOAC and the reason for this selection.
- We recorded the frequency of clinical follow-up visits, as well as the frequency and type of follow-up blood-testing for patients treated with NOACs.
- We assessed the frequency of bleeding complications encountered in general practices in the preceding two years, as well as the type and localisation of bleeding complications and the need for hospital admission.

All questionnaires were anonymised before analysis. All participating GPs gave their written consent for the analysis and publication of collected data.

As no individual patient data are acquired in our survey, no ethical approval is necessary.

We performed all statistical calculations with SPSS Statistics 21 (IBM Corp.) and used descriptive statistics as appropriate.

## Results

### Demographic characteristics

53 GPs participated in our survey (response rate 40.8%). The mean age was 49.3 ± 10.8 years (SD, range 28–67 years) with a working experience of 13.7 ± 11.3 years (range 1–40 years). 26 GPs were female (49.1%) and 27 male (50.9%). Four GPs had no speciality (7.5%); 49 (92.5%) were specialists in general internal medicine. In Switzerland, after successfully completing general practitioner training, the title “FMH Allgemeine Innere Medizin” (specialist in general internal medicine) is awarded.

### NOACs versus VKAs

New patients who were started on oral anticoagulation received NOACs and not VKAs from 49 GPs (92.5%), unless there were contraindications or patient preferences that were independent of the indication for anticoagulation. The preference for the prescription of NOACs in comparison to VKAs are shown in Table 1 for specific indications. The reasons for starting directly with NOACs are listed in Table 2. All GPs included in our survey treat patients with NOACs (n = 53). Altogether GPs in our survey treated 32.7% ± 19.0 (range 10–80%) of their patients on oral anticoagulation with NOACs. In patients already under therapy with VKAs, 14 GPs (26.4%) would decide to switch to any NOAC. Eight GPs (15.1%) indicated that they were not prepared to switch stable patients and five GPs (9.4%) would not switch because of the higher costs of NOACs.

**Table 1:** Preference for NOACS to VKA by indication

Indication	Number (%)
Prevention of recurrent PE/DVT	43 (81.1%)
Treatment of PE/DVT	42 (79.2%)
Prophylactic post-surgery	39 (73.6%)
Atrial fibrillation	37 (69.8%)

n = 53. Multiple answers possible. Absolute number (%)

*PE* pulmonary embolism, *DVT* deep venous thrombosis

**Table 2:** Reasons for direct start with NOACs instead of VKAs

Reasons	Number (%)
Easier dosing	51 (96.2%)
Fewer blood tests	48 (90.6%)
Fewer clinical follow-ups	33 (62.3%)
Fewer bleeding events	24 (45.3%)
Age of patients	20 (37.7%)
Better tolerance	5 (9.4%)

Multiple answers possible. n = 53. Absolute number (%)

### NOAC preference

Rivaroxaban is the NOAC prescribed by most GPs (75.5%). In general, 52 GPs (98.1%) prescribed rivaroxaban, 8 apixaban (15.1%), 6 dabigatran (11.3%) and 3 edoxaban (5.6%). The reasons for these preferences are shown in Table 3.

**Table 3:** Reasons for NOAC preference

Reasons	Number (%)
Prescription by specialists	37 (69.8%)
Evidence	27 (50.9%)
Prevalence	25 (47.2%)
Patient’s profile	13 (30.2%)
Easier dosing OD	9 (17.0%)
Patient’s choice	7 (13.2%)
Personal experience	3 (5.7%)

Multiple answers possible. n = 53. Absolute number (%)

### Follow-up of NOAC

The frequency of clinical follow-up consultations and follow-up blood tests of patients with NOACs are reported in Table 4. Clinical controls are scheduled by 36 of the GPs (67.9%) at least every 3 months. Creatinine and haemoglobin are laboratory parameters monitored by the majority of GPs (51 (96.2%) and 39 (73.6%), respectively).

**Table 4:** Frequency of clinical follow-up consultations and blood tests of patients treated with NOACs

Frequency	Clinical	Creatinine	INR	Liver enzymes	Haemoglobin
Never	2 (3.8%)	2 (3.8%)	46 (86.8%)	21 (39.6%)	14 (26.4%)
Once	0 (0%)	3 (5.7%)	3 (5.7%)	2 (3.8%)	3 (5.7%)
Monthly	1 (1.9%)	2 (3.8%)	0 (0%)	2 (3.8%)	2 (3.8%)
Every 2–3 months	35 (66%)	17 (31.2%)	1 (1.9%)	4 (7.5%)	11 (20.8%)
Every 6 months	13 (24.5%)	21 (39.6%)	0 (0%)	10 (18.8%)	15 (28.3%)
Annually	2 (3.8%)	8 (15.1%)	3 (5.7%)	14 (26.4%)	8 (15.1%)

n = 53

*INR* International normalised ratio

### Bleeding complications

In the preceding 2 years, GPs had seen 1.9 ± 2.87 (range 0–14) bleeding complications in patients with NOACs in their practice. For details of the bleeding complications in patients with NOACs, see Table 5. The GPs had to refer 0.5 ± 0.95 (range 0–5) of these patients to hospital treatment.

**Table 5:** Overview of bleeding complications of patients treated with NOACs

Bleeding complications	Absolute number/year	Number/GP/year
*Severity*		
Minor	31	0.58
Life-threatening	13.5	0.25
*Location*		
Epistaxis	17	0.32
Haematoma	12	0.23
Gastrointestinal bleeding	11.5	0.22
Intracranial bleeding	2	0.04
Abdominal bleeding	0	0
Other	2	0.04

Multiple answers possible. n = 53

## Discussion

In the present study, we aimed to analyse everyday use and experiences with NOACs in general practise.

### NOACs versus VKAs

The growing importance of NOACs is broadly reflected in the daily practice of Swiss GPs surveyed in our investigation. Nearly all new patients with a new indication for oral anticoagulation are started on NOACs, although international guidelines differ. In contrast to the guidelines from the European Society of Cardiology (ECS), the American Heart Association (AHA) guideline for the management of patients with AF still recommends VKAs rather than NOACs (January et al. 2014; Camm et al. 2012).

It is striking that “atrial fibrillation” (AF)—the indication for which NOACS were first approved—is the least preferred indication in our dataset. In venous thrombosis, NOACs are more broadly accepted by the GPs in our survey, although the corresponding guideline by the ESC gives no specific recommendation (Konstantinides et al. 2014). To facilitate the choice between NOACs and VKAs in the future, GPs have the possibility of using newer decision tools like the SAMe-TT2R2 score to predict poor INR outcome (Proietti and Lip 2015).

In contrast to new patients started on oral anticoagulation, existing therapy with VKAs was mostly continued in our population. This is consistent with the recommendations in the AHA guidelines not to switch stable and satisfied patients on easily controlled VKA therapy (January et al. 2014).

### NOAC preference

Because no head-to-head trials of NOACs exist, there are no clear recommendations for the choice of a specific NOAC. By far the most common reason given in our survey was a pre-existing prescription by a specialist or hospital.

### Follow-up of patients with NOAC

About two-thirds of GPs clinically monitor their patients on NOACs at least every 3 months, even though INR testing is no longer necessary, as it is for patients under VKAs. This complies with the only guideline available at the moment: the practical guideline from the European Heart Rhythm Association (EHRA). This guideline recommends clinical follow-ups initially after 1 month and later every third month (Heidbuchel et al. 2015). About the same percentage of GPs follow the recommendations for blood tests: The EHRA guideline recommends annual haemoglobin and liver enzyme controls. The recommendation for creatinine controls depend on renal function and we did not differentiate this in our questionnaire. In general, creatinine is checked by the GPs in our survey more frequently than other blood parameters, as requested in the EHRA guideline. This is reassuring, giving the fact that in an American survey Simon et al. (2015) found that creatinine was not measured in about 40% of patients, not even at the time of prescription.

However, given the potentially dangerous complications in patients on oral anticoagulation, an overall adherence rate to current follow-up guidelines of only about two-thirds seems suboptimal and should prompt further research and would need improvement. This might also include a comparison of real-life follow-up consultations and blood tests in NOAC versus VKA patients.

### Bleeding events

The type of bleeding complications and the ratio of minor to major bleeding events seen in our survey matches the results of a previous study of our ED patients (Sauter et al. 2016). Minor bleeding complications (epistaxis, haematoma) were encountered about every second year by a GP, and major bleeding complications (gastrointestinal bleeding, intracranial or abdominal bleeding) about every fourth year. Furthermore, very few patients have to be referred to a hospital because of bleeding complications. It is important to keep in mind that patients with major bleeding may present directly to hospital emergency departments and may not consult their GP. Therefore the absolute number of major bleeding events is likely to be underestimated by the GPs. The hospitalisation rate seems especially important for the comparison of costs in NOACs versus VKAs, as hospitalisation because of a bleeding event is a major cost factor (Kim et al. 2010; Ghate et al. 2011). For this purpose, a comparison of NOAC versus VKA for GP reported bleeding events would also be of interest in follow-up investigations.

### Limitations

The sample of GPs in our survey is very small and may not be representative, because we conducted the survey as a pilot study in unselected GPs presenting to a congress. It is conceivable that GPs presenting to an emergency medicine congress are more progressive and educated, as well as being more aware of emergency medicine and complications than average. We did not ask GPs how many patients they cared for and therefore cannot calculate a risk per patient in bleeding complications. Our pilot study demonstrates the areas of knowledge in the handling of NOACs by GPs that need further research. In particular, future studies should focus on adherence to the guidelines from the European Society of Cardiology and the American Heart Association and on the control of patients’ adherence to the treatment. This first pilot investigation can only provide initial insights and might promote awareness of real-life problems and stimulate further research.

## Conclusions

On the basis of our pilot survey, NOACs seem broadly accepted among the investigated Swiss GPs as first choice—in preference to VKAs—for patients newly requiring oral anticoagulation, especially if recommended by haematologists or cardiologists. Because only about two-thirds of GPs in our survey adhere to recommendations on clinical and blood test follow-ups, further efforts to implement follow-up guidelines seem necessary, as is further extended research in a large representative GP population—including a comparison of NOACs and VKAs. Bleeding complications reported by our population are rare and were mostly handled in the outpatient setting without hospital admission.

## Abbreviations

AF: atrial fibrillation
ED: emergency department
ESC: European Society of Cardiology
GP: general practitioner
NOACs: new direct oral anticoagulants
VKAs: vitamin K antagonists
